# Risk factors for fatal candidemia caused by *Candida albicans *and non-albicans *Candida *species

**DOI:** 10.1186/1471-2334-5-22

**Published:** 2005-04-07

**Authors:** Ming-Fang Cheng, Yun-Liang Yang, Tzy-Jyun Yao, Chin-Yu Lin, Jih-Shin Liu, Ran-Bin Tang, Kwok-Woon Yu, Yu-Hua Fan, Kai-Sheng Hsieh, Monto Ho, Hsiu-Jung Lo

**Affiliations:** 1 Department of Pediatrics, Veterans General Hospital-Kaohsiung, Kaohsiung, Taiwan; 2 Section of Infection Disease, Department of Pediatrics, Taiwan; 3 Department of Microbiology, Veterans General Hospital-Taipei, Taiwan; 4 National Yang Ming University, Taipei, Taiwan; 5 Department of Biological Science and Technology, National Chiao Tung University, Hsinchu, Taiwan; 6 Division of Biostatistics and Bioinformatics, Miaoli, Taiwan; 7 Koahsiung Medical University, Kaohsiung, Taiwan; 8 Division of Clinical Research, National Health Research Institutes, Miaoli, Taiwan

## Abstract

**Background:**

Invasive fungal infections, such as candidemia, caused by *Candida *species have been increasing. Candidemia is not only associated with a high mortality (30% to 40%) but also extends the length of hospital stay and increases the costs of medical care. Sepsis caused by *Candida *species is clinically indistinguishable from bacterial infections. Although, the clinical presentations of the patients with candidemia caused by *Candida albicans *and non-albicans *Candida* species (NAC) are indistinguishable, the susceptibilities to antifungal agents of these species are different. In this study, we attempted to identify the risk factors for candidemia caused by *C. albicans *and NAC in the hope that this may guide initial empiric therapy.

**Methods:**

A retrospective chart review was conducted during 1996 to 1999 at the Veterans General Hospital-Taipei.

**Results:**

There were 130 fatal cases of candidemia, including 68 patients with *C. albicans *and 62 with NAC. Candidemia was the most likely cause of death in 55 of the 130 patients (42.3 %). There was no significant difference in the distribution of *Candida *species between those died of candidemia and those died of underlying conditions. Patients who had one of the following conditions were more likely to have *C. albicans*, age ≧ 65 years, immunosuppression accounted to prior use of steroids, leukocytosis, in the intensive care unit (ICU), and intravascular and urinary catheters. Patients who had undergone cancer chemotherapy often appeared less critically ill and were more likely to have NAC.

**Conclusion:**

Clinical and epidemiological differences in the risk factors between candidemia caused by *C. albicans *and NAC may provide helpful clues to initiate empiric therapy for patients infected with *C. albicans *versus NAC.

## Background

Invasive fungal infections caused by *Candida *species have increased significantly. They now rank fourth as the most common cause of nosocomial bloodstream infections in the United Stated [1] and the most common one at one major hospital in Taiwan [2]. Candidemia is not only associated with a mortality of about 30% to 40% but also extends the duration of hospital stay [3,4] and increases the cost for medical care [5]. In recent years, *Candida *species associated with candidemia have shifted from *Candida albicans *to non-albicans *Candida *species (NAC) [6]. Approximately half of the reported cases of candidemia are now caused by NAC [7-10].

Patients with candidemia usually present an acute septic syndrome that is indistinguishable from bacteremia, but they may also exhibit a more indolent course manifested by fever of unknown origin. Major risk factors for candidemia include intravascular catheters, parenteral hyperalimentation, and broad-spectrum antibiotics. Empirical antifungal agents may be administrated to patients manifested with fever of unknown origin and have above mentioned risk factors, particularly those who have been treated with broad-spectrum antibiotics. The clinical presentation of the patients with sepsis caused by *C. albicans *and NAC are indistinguishable. However, NAC are often less susceptible to fluconazole than *C. albicans *is [7,11,12] and may require greater dosage to cure clinically [13,14].

We have conducted a retrospective chart review of patients whose death was associated with candidemia. The goal was to assist decisions to select the most appropriate empirical therapy for patients suspected to have candidemia. The objective was to determine whether specific risk factors could be identified to help selecting those patients who are more likely infected with *C. albicans *versus NAC.

## Methods

This study was conducted at the Veterans General Hospital-Taipei. This is a 2800-bed teaching hospital with about 69,000 admissions annually [7]. A total of 415 blood isolates of *Candida *species (one strain per species per patient) were collected from April 1996 to December 1999. During this period 130 patients met the inclusion criteria of having at least one positive blood culture for *Candida *species within one month prior to the fatal outcome. There were 68 patients infected with *C. albicans *and 62 with NAC.

*Candida *species were isolated from blood using BACTEC 860 system (Becton Dickinson, INC, Sparks, MD). The species were identified using API-32C system (bioMerieux Vitek, Inc, St. Louis, MI).

### Clinical data

Clinical data of 130 patients were recorded on standardized forms and analyzed according to age at the time of diagnosis, gender and underlying illnesses. These included cancer, diabetes, immunosuppression accounted to prior use of steroids, systemic antifungal agents, cancer chemotherapy, parenteral nutrition, the presence of central or arterial line, endotracheal tube, and urinary catheter, admission to an intensive care unit (ICU), history of infection or gastrointestinal surgery, hemogram, antifungal management, and cause of death. The predisposing events within one month prior to diagnosis and the laboratory data within seven days prior to obtaining the first positive blood culture were analyzed. To decide that a patient died directly of candidemia or underlying illness, we recoded the causes of death according to the death certificate of the patients.

### Statistical analysis

The statistical significance of association between categorical variables was assessed using the Fisher's Exact Test. The risk factors with p values < 0.1 in the univariate analysis were placed in a multivariate analysis using the multiple logistic regression package in the SAS System for Windows V8. Confidence limit of 95% means that the confidence limit contains the true value of odds ratio with probability of 0.95. The narrower the confidence limit is, the more accurate the estimate is.

## Results

The population consisted of 130 patients whose death was associated with candidemia. Fifty-five (42.3 %) of these deaths were considered to be directly caused by *Candida *species. The remaining died of complications of their underlying illness. The distribution of species of the total deaths and those causally related to candidemia is shown in Table 1. There were no significant differences in causation of death according to species. Interestingly, of the four major species related to candidemia, 61.5% of the isolated *Candida glabrata *contributed to mortality, followed by 50% of *Candida parapsilosis*, 41.2% of *C. albicans*, and 31.3% of *Candida tropicalis*.

**Table 1:** Distribution of *Candida species *of fatal outcome attributed to disseminated candidemia

	Blood isolates (N = 130)		Death directly related to candidemia (N = 55)		
		
species	% in Number 130 isolates		% in Number 55 isolates		% in species of blood isolates
*C. albicans*	68	52.3	28	50.9	41.2
non-albicans *Candida *species	62	47.7	27	49.1	43.5
*C. tropicalis*	32	24.7	10	18.2	31.3
*C. glabrata*	13	10.0	8	14.5	61.5
*C. parapsilosis*	12	9.2	6	10.9	50.0
*C. guilliermondii*	2	1.5	1	1.8	50.0
*C. peniculosa*	2	1.5	2	3.6	100.0
*C. famata*	1	0.8	0	0	0

The demographic and clinical characteristics of the study population categorized according to the presence of *C. albicans *or NAC in the blood are summarized in Table 2. The significant (*p *≤ 0.05) risk factors on univariate analysis for *C. albicans *candidemia were age ≧ 65 years, prior bacterial urinary tract infection, central venous catheter, parenteral nutrition and leucocytosis with white blood cell counts (WBC) ≧ 15000/mm^3^, without significant neutropenia with absolute neutrophil counts (ANC) ≧ 100000/mm^3^. The significant risk factors for NAC were age < 65 years, cancer chemotherapy, neutropenia (WBC < 3000/mm^3^) and severe thrombocytopenia (platelet count ≦ 20000 /mm^3^). NAC patients were more likely to have cancers and to be located on the medical wards (p = 0.08). When multivariate analysis was employed, the significant risk factors for *C. albicans *were age ≧ 65 years, immunosuppressive therapy, and leucocytosis. Significant risk factors for NAC were age < 65 years and location on medical wards, which are shown in Table 3.

**Table 2:** Characteristics of 130 patients who died in association with candidemia

Characteristic	*C. albicans *(N = 68)		NAC (N = 62)		Fisher's Exact Test
	No.	%	No.	%	Two-sided P-value
	
Age ≧ 65 years old	56	82.4	36	58.1	0.004*
MICU	6	8.8	10	16.1	0.286
Medical ward stay	24	35.3	32	51.6	0.077*
Cancer	24	35.3	32	51.6	0.077*
Immunosuppressive therapy	17	25	7	11.3	0.069*
Bacteremia	25	36.8	15	24.2	0.133
Bacterial urinary tract infection	23	33.8	10	16.1	0.026*
Prior antifungal administration	5	7.4	9	14.5	0.259
Catheters within 30 days	65	95.6	56	90.3	0.309
ETT	35	51.5	23	37.1	0.114
CVC	57	83.8	41	66.1	0.025*
AL	21	30.9	14	22.6	0.326
Urinary catheter	49	72.1	39	62.9	0.348
PPN	50	73.5	34	54.8	0.029*
Cancer chemotherapy	5	7.4	12	19.4	0.066*
Corticosteroids	26	38.2	18	29	0.354
ANC	(N = 44)		(N = 40)		1
ANC ≦ 1500/mm^3^	2	4.5	2	5	
WBC	(N = 67)		(N = 61)		0.0004*
≦ 3000/mm^3^	4	6	14	23	
3000/mm^3 ^to 5000/mm3	35	52.2	38	62.3	
> 15000/mm^3^	28	41.8	9	14.8	
Platelet	(N = 66)		(N = 61)		0.011*
≦ 20000/mm^3^	5	7.6	10	16.4	
20000/mm^3 ^to 100000/mm^3^	20	30.3	29	47.5	
> 100000/mm^3^	41	62.1	22	36.1	

* Factors selected for multivariate analysis. AL, arterial line; ANC, absolute neutrophil count; CVC, central venous catheter; ETT, endo trachial tube; ICU, Intensive care unit; PN, parenteral nutrition; WBC, white blood cell

**Table 3:** Multiple regression analysis of risk factors for candidemia caused by *Candida albicans *vs. non-albicans Candida species

Effect	Odds Ratio	95% confidence Limits		P-value
		
White Blood Cell (WBC)				0.0202
> 15000/mm^3 ^vs. ≦ 3000/ mm^3^	5.89	1.35	25.7	0.0184
3001 – 15000/mm^3 ^vs. ≦ 3000/ mm^3^	1.66	0.44	6.26	0.4549
> 15000/mm^3 ^vs. 3001 – 15000/mm^3^	3.55	1.29	9.79	0.0144
Age65 (≧ 65 vs. < 65)	6.73	2.37	19.1	0.0003
Immunosuppressive therapy	4.39	1.26	15.32	0.0201
Located on a medical ward	0.37	0.16	0.88	0.025

## Discussion

*Candida albicans *remains the most common species causing candidemia, but the proportion caused by NAC is increasing [7-10]. Patients with NAC are more likely to require greater dosage of fluconazole to cure clinically [13,14]. Thus there is a need to identify patients at risk of NAC candidemia to initiate empirical amphotericin B therapy or high-dose fluconazole.

Krcmery and Barnes have identified the following risk factors for NAC [15]. These include prophylaxis with azole compounds as a risk factor for *C. krusei *and *C. glabrata*, neutropenia and bone marrow transplantation for *C. tropicalis*, and insertion of foreign bodies, neonates, and hyperalimentation for *C. parapsilosis *[15-18]. In this study we have identified additional risk factors that help distinguish candidemia caused by *C. albicans *and NAC. The most important risk factors for *C. albicans *are the old age, procedures associated with intensive care, and an acute sepsis. The most important risk factors for NAC are cancer chemotherapy in association with leukopenia and thrombocytopenia.

It has been reported that *Candida *species exhibit a spectrum in the extent of adherence to tissues, which correlates with the pathogenicity in humans and animals [19]. *Candida albicans *exhibit the greatest capacity to adhere to gingival epithelial cells, followed by *C. tropicalis *and *C. glabrata *[19]. The capacity of yeasts to attach to a wide range inanimate surfaces appears to protect them from immune responses and antimicrobial agents [20]. This may explain why *C. albicans *is more likely to be associated with central venous catheters in this study.

Conventional methods to eradicate *C. albicans *rely upon the use of antifungal drugs designed to kill the yeast or arrest its growth. However, removal of intravascular catheters is in fact the most simple and effective method [21,22]. Another approach is to design new agents that disrupt adherence of the yeasts to host tissues and catheters.

## Conclusion

It is customary to administer amphotericin B for critically ill patients suspected of having candidemia. More often than not, azoles are used for relatively stable patients. NAC tend to be less susceptible to fluconazole than *C. albicans *is [7,11,23]. Thus, greater dosage is needed till the causative microorganism is isolated and identified, particularly with patients at high risk for NAC candidemia. Though, a large, prospective study is needed to validate this concept, the risk factors identified in this study may help, in clinical practice, to differentiate fatal candidemia caused by *C. albicans *versus NAC.

## Abbreviations used

NAC, non-albicans *Candida *species; VGH-TPE, Veterans General Hospital-Taipei; ICU, intensive care unit; AL, arterial line; ANC, absolute neutrophil count; CVC, central venous catheter; ETT, endo trachial tube; WBC, white blood cell.

## Competing interests

The author(s) declared that they have no competing interests.

## Authors' contributions

MFC conceived the study and designed it together with, YLY, MH, and HJL. MFC conducted the experiments with contribution from RBT, YHF, and KSH. KWY collected clinical isolates. JSL performed the statistical study with contributions from CYL and TJY. MFC drafted the manuscript with contribution from YLY and HJL.

## Pre-publication history

The pre-publication history for this paper can be accessed here:


